# Construction and validation of an ultrasound-based nomogram model for predicting dysphagia in patients with chronic obstructive pulmonary disease

**DOI:** 10.3389/fmed.2025.1533165

**Published:** 2025-07-08

**Authors:** Shanshan Su, Qichen Su, Huohu Zhong, Guorong Lyu, Yuanzhe Li, Yi Wang, Zhirong Xu

**Affiliations:** ^1^Department of Ultrasound in Medicine, Second Affiliated Hospital of Fujian Medical University, Quanzhou, China; ^2^Department of CT/MRI, Second Affiliated Hospital of Fujian Medical University, Quanzhou, China

**Keywords:** chronic obstructive pulmonary disease, columnar graphical modeling, dysphagia, ultrasound, prediction

## Abstract

**Introduction:**

Dysphagia is common in chronic obstructive pulmonary disease (COPD), prompting the need for predictive models for this condition. In this study, we aimed to develop a nomogram model for dysphagia prediction in patients with COPD.

**Methods:**

Data from 300 patients with COPD were divided into the training (*n* = 210) and validation (*n* = 90) cohorts. Independent risk factors for dysphagia were identified using logistic regression and used to construct a nomogram model. The model’s predictive efficacy, accuracy, and clinical utility were evaluated using receiver operating characteristic curve analysis, calibration, decision curve analysis, and clinical impact curves.

**Results:**

Hypoglossal-hyoid shortening rate, hyoid-larynx shortening approximation distance, chin time of movement of the genioglossus, and distance of movement of the genioglossus were identified as independent risk factors. The nomogram exhibited areas under the curve of 0.834 and 0.804 in the training and validation cohorts, respectively, indicating good predictive efficacy and calibration.

**Conclusion:**

The nomogram model effectively predicts dysphagia occurrence in patients with COPD, providing a valuable tool for risk assessment.

## 1 Introduction

Chronic obstructive pulmonary disease (COPD) is a prevalent chronic respiratory condition in clinical practice, characterised by persistent respiratory symptoms, airflow limitation, and abnormal inflammatory responses. This disorder is typically triggered by smoking or exposure to harmful particles (1). Chronic respiratory disturbances caused by COPD may extend beyond the respiratory system and affect swallowing function (2, 3).

Swallowing, under normal conditions, involves a series of coordinated movements initiated by a complex network of sensory receptors, facilitating food intake through the upper respiratory/gastrointestinal tract. Governed by the brain, this process requires synchronized participation of the mouth, pharynx, and oesophagus. Dysfunction in any of these areas can lead to dysphagia, presenting an added challenge in managing COPD (4).

Stable and coordinated interactions between respiration and swallowing are crucial in adults. During normal swallowing, a brief and well-timed pause is observed in breathing known as the swallow apnoea, which prevents food or liquid from entering the airway. This coordinated pause in breathing is crucial for protecting the airway and ensuring the safe passage of ingested material into the digestive system (5, 6). However, patients with COPD often exhibit reduced coordination owing to dysfunctional upper respiratory tract protective mechanisms and altered breathing habits (7). Thus, the aspiration rate in patients with stable COPD can be as high as 25%, with dysphagia being the primary cause (8). In a typical swallowing process, the airflow dynamics ensure that food does not inadvertently enter the airway, since the lung pressure exceeds that of the airway opening. Disruption of this normal expiratory-swallowing-expiratory pattern (such as swallowing during the inspiratory phase) can lead to food being inadvertently inhaled into the lungs, facilitated by negative inspiratory pressure. This inhalation of foreign matter, particularly when bacteria are present, can lead to bronchitis, bronchiolitis, or pneumonia, suggesting that dysphagia may be a risk factor for acute exacerbation in patients with COPD (9).

The videofluoroscopic swallowing study (VFSS) is the most widely used method for diagnosing dysphagia (10). This method utilises fluoroscopy to observe the swallowing process, providing insight into functional and anatomical abnormalities in the process and enabling comprehensive evaluation. Despite clear grading and easy operation being obvious advantages of VFSS, this procedure has limitations, including the risk of barium aspiration, inability to repeat the procedure, inability to assess pharyngeal sensory function, and high radiation exposure. Moreover, VFSS requires specialized equipment and is unsuitable for critically ill patients, those with impaired consciousness, aphasia, or patients who are unable to cooperate (11). In addition, dysphagia symptoms may fluctuate, necessitating ongoing monitoring and intervention. Consequently, while VFSS is valuable, its limitations hinder its applicability for bedside examination and continuous monitoring in clinical practice.

Under-chin ultrasonography is a non-invasive, cost-effective, and rapid modality for evaluating swallowing disorders (12). Unlike fluoroscopy, it poses no radiation risk and permits repeatability. Direct visualization of tongue and hyoid bone dynamics through ultrasound images offers valuable clinical insights. Rocha et al. (13) quantified tongue advancement time and maximum hyoid bone displacement during the oropharyngeal phase, demonstrating the potential of ultrasound in dysphagia assessment and in therapeutic feedback. Ming et al. (14) characterized hyoid bone movement trajectories during swallowing, noting reduced tongue muscle thickness and hyoid bone displacement in patients with dysphagia. This study employed ultrasonography to establish diagnostic thresholds and objectively identify high-risk patients.

Nomogram is a graphical representation of a mathematical formula or model designed to predict a specific outcome. It is a tool commonly used in various fields such as medicine, statistics, and engineering to make complex calculations more accessible and user-friendly. Nomograms typically consist of a set of scales or lines representing different variables involved in the prediction model. By drawing a straight line between values on one scale to values on another and then projecting it to the outcome scale, one can determine the probability or outcome being predicted. In the medical field, nomograms are often used for predicting risks of certain diseases, prognosis of patients, treatment outcomes, or other clinical parameters. They are particularly useful for individualized risk assessment, as they allow healthcare professionals to estimate probabilities based on specific patient characteristics without performing complex calculations each time (15).

In this study, we aimed to identify risk factors associated with dysphagia occurrence, construct a predictive model, and visualize it using a nomogram to enhance its clinical utility. To our knowledge, this is the first study to construct a nomogram for visualising dysphagia risk.

## 2 Methods

### 2.1 Patients

Data were collected between January 2022 and December 2023 from 300 patients with COPD at the Second Affiliated Hospital of Fujian Medical University. This study was conducted in accordance with the tenets of the Declaration of Helsinki and approved by the Ethics Committee of the Second Affiliated Hospital of Fujian Medical University.

The inclusion criteria were as follows: (1) diagnosis of stable COPD according to the GOLD Association 2023 guidelines (16), with no previous history of acute exacerbation; (2) age of 40–89 years and conscious; and (3) stable vital signs without serious complications in the heart, lungs, kidneys, and other organs.

The exclusion criteria were as follows: (1) inability to maintain a seated position or to keep the head in a neutral position; (2) previous history of pharyngeal surgery or other diseases affecting swallowing function, along with a history of severe injuries to vital organs, and inability to tolerate strong stimuli, such as lifting or choking; (3) localized pharyngeal disorders; (4) history of brain injury or stroke; (5) recent receipt of dysphagia treatment or use of drugs affecting swallowing function within the last 3 months, such as muscle relaxants or diuretics; and (6) cognitive impairment or consciousness disorder hindering cooperation for completing the test.

### 2.2 Ultrasound examination

#### 2.2.1 Ultrasound instrument

The Mindray R7 ultrasound diagnostic instrument equipped with convex array probe set at frequencies of 2–5 MHz and a line array probe set at frequencies of 7–12 MHz was used.

#### 2.2.2 Ultrasound scanning

**Figure 1:**
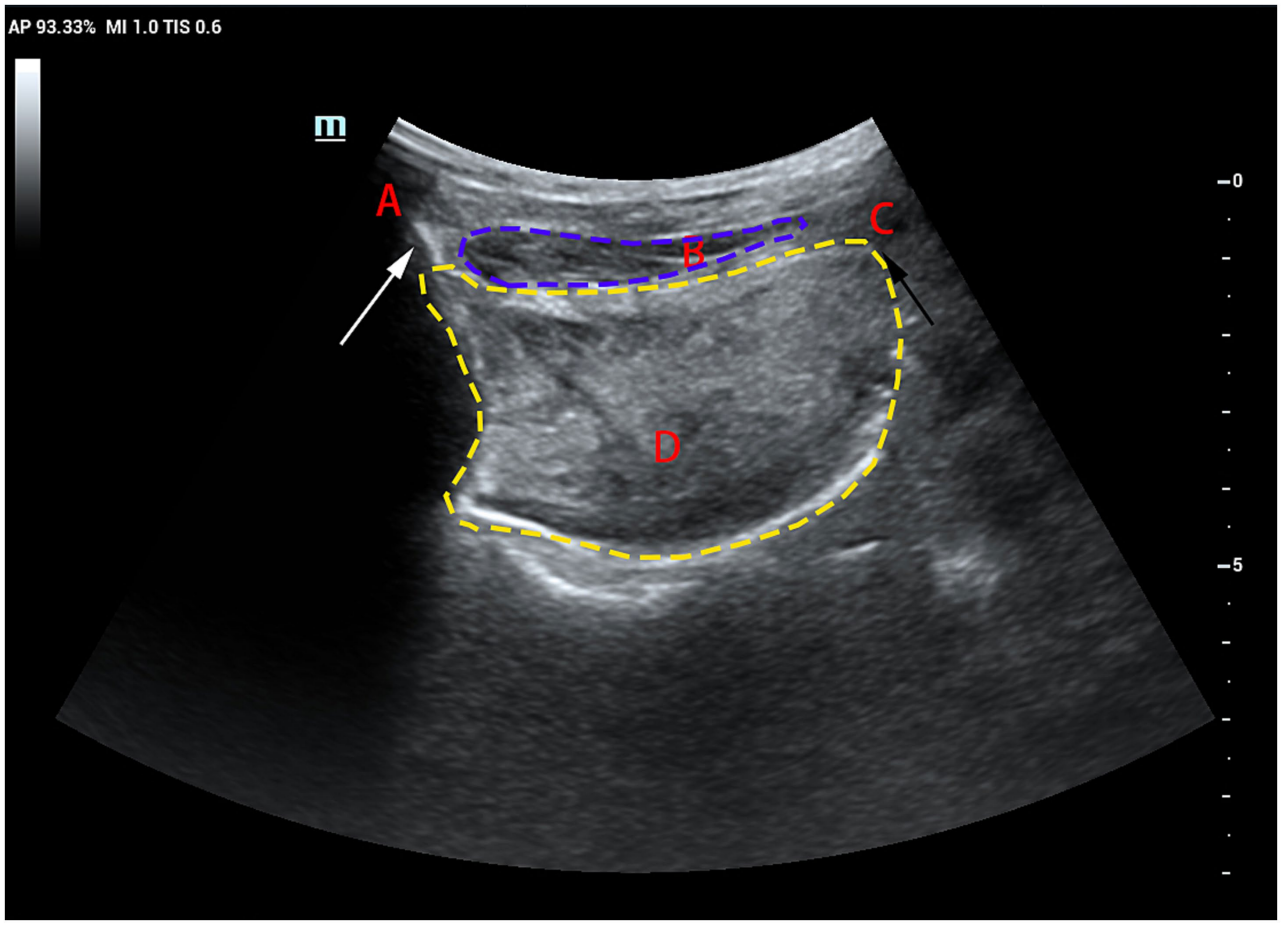
B-mode ultrasound image showing the genioglossus and the hyoid bone in the median sagittal position **(A)** mandible: as indicated by the white arrow; **(B)** genioglossus: as indicated by the blue dotted curve; **(C)** hyoid: as indicated by the black arrow; **(D)** tongue: as indicated by the yellow dotted curve.

**Figure 2:**
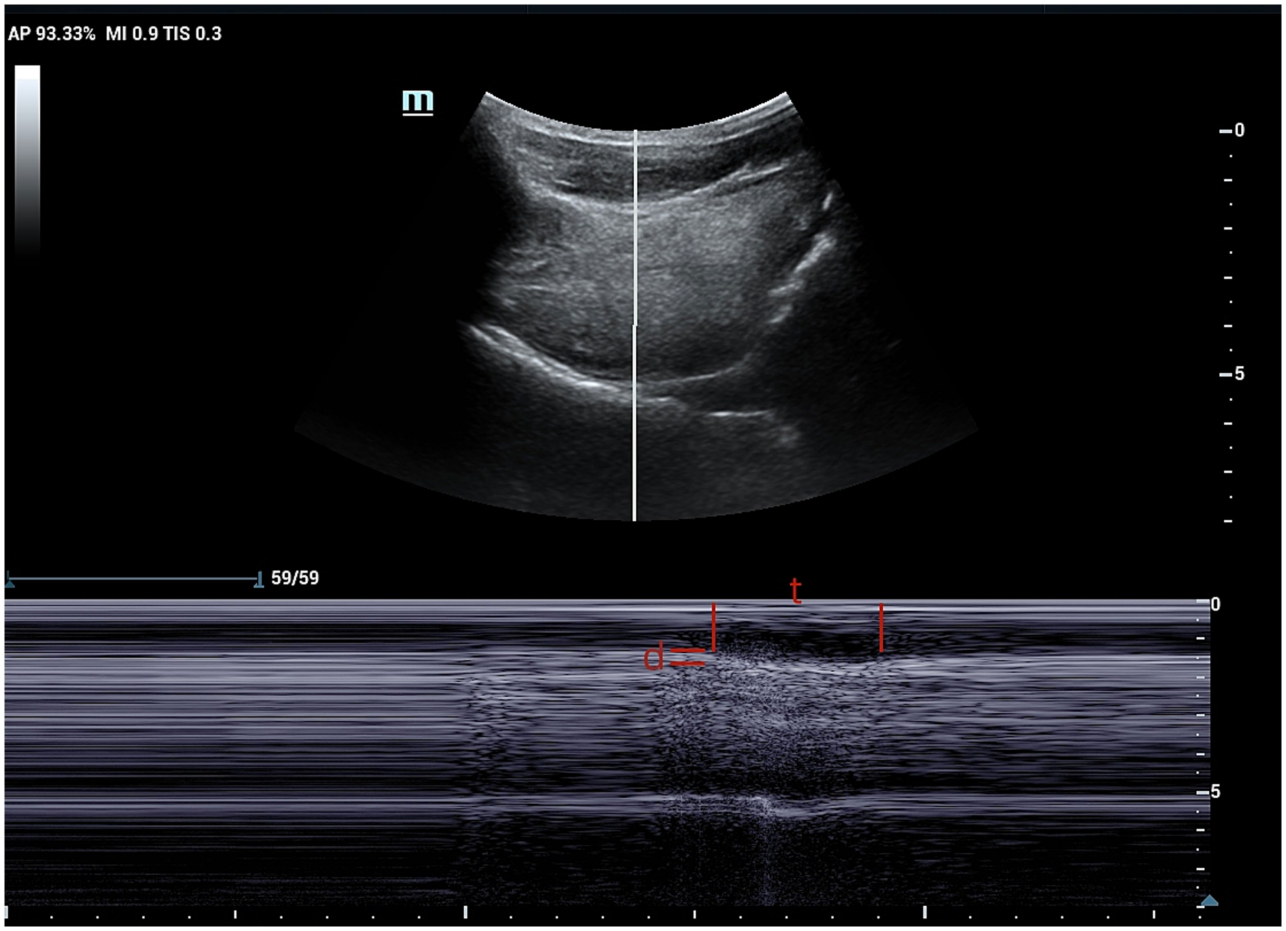
M-mode ultrasound image showing time and distance of genioglossus movement. The sampling line is placed at 2 cm from the hyoid bone. t, time of genioglossus movement; d, distance of genioglossus movement.

**Figure 3:**
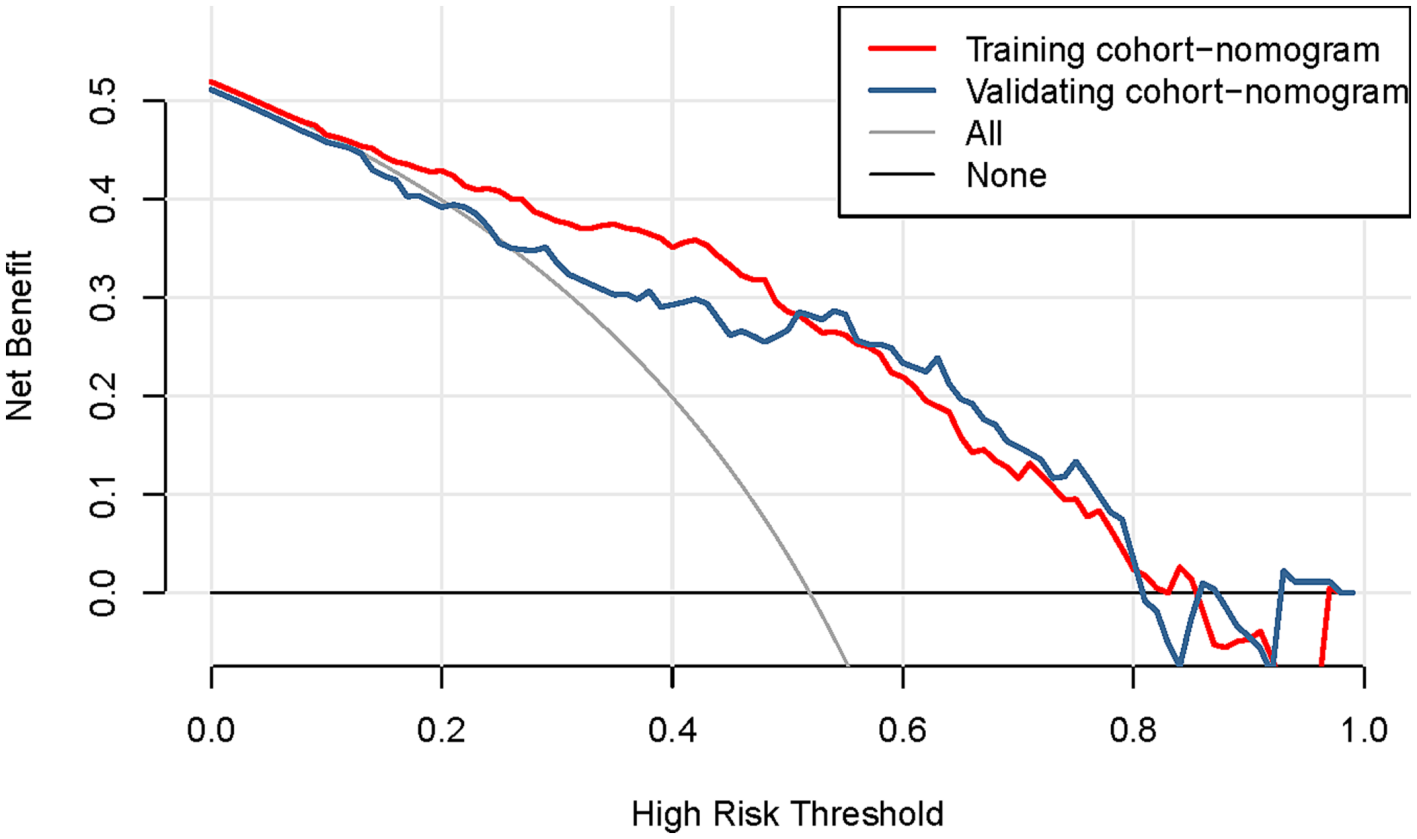
B-mode ultrasound image of hyoid-larynx approximation shortening distance.

Patients were seated upright on a reclining chair with their heads resting against the chair’s back in a neutral position. The examiner applied ample ultrasound coupling agent to the laryngeal skin and gently manoeuvred the probe to prevent interference with swallowing. The probe was positioned parallel to the median sagittal position plane of the larynx. First, a clear image of the chin-hyoid muscle and hyoid bone median sagittal plane was obtained (Figure 1). Then, utilising B/M-type imaging, the sample line was positioned 2 cm from the angle of the hyoid bone (Figure 2). Patients were instructed to swallow 5 mL of mineral water (24–26°C); during this process, dynamic swallowing images were captured, showing the longitudinal section from the tongue root to the thyroid cartilage, where the hyoid bone and thyroid cartilage appeared as two posteriorly accompanied structures with acoustic shadowing. The distance between these structures represented the Hyolaryngeal Apparatus (HLA) (Figure 3). The examination was repeated three times in each patient, with a 2-min interval between each to prevent fatigue.

#### 2.2.3 Evaluation of ultrasound results

Recorded images were analyzed frame-by-frame to determine the maximum change in hyoid muscle thickness and calculate the change value. Additionally, M-mode ultrasound was used to measure chin-hyoid muscle movement distance and time. Hyoid bone movement parameters, including maximum and minimum hyoid-larynx approximation (MHLA and NHLA), were measured through frame-by-frame playback. Hyoid-larynx approximation shortening (HLAS) distance was computed as the difference between MHLA and NHLA, while the hyoid-larynx shortening rate (ASR) was calculated as HLSA divided by MHLA, multiplied by 100%.

#### 2.2.4 Ultrasonic image quality control

The implementation of ultrasound examination is completed by a highly trained sonographer, and the obtained ultrasonogram would be submitted to the sonographer with the title of associate senior or above for review.

#### 2.2.5 Diagnosis of dysphagia

**Figure 4:**
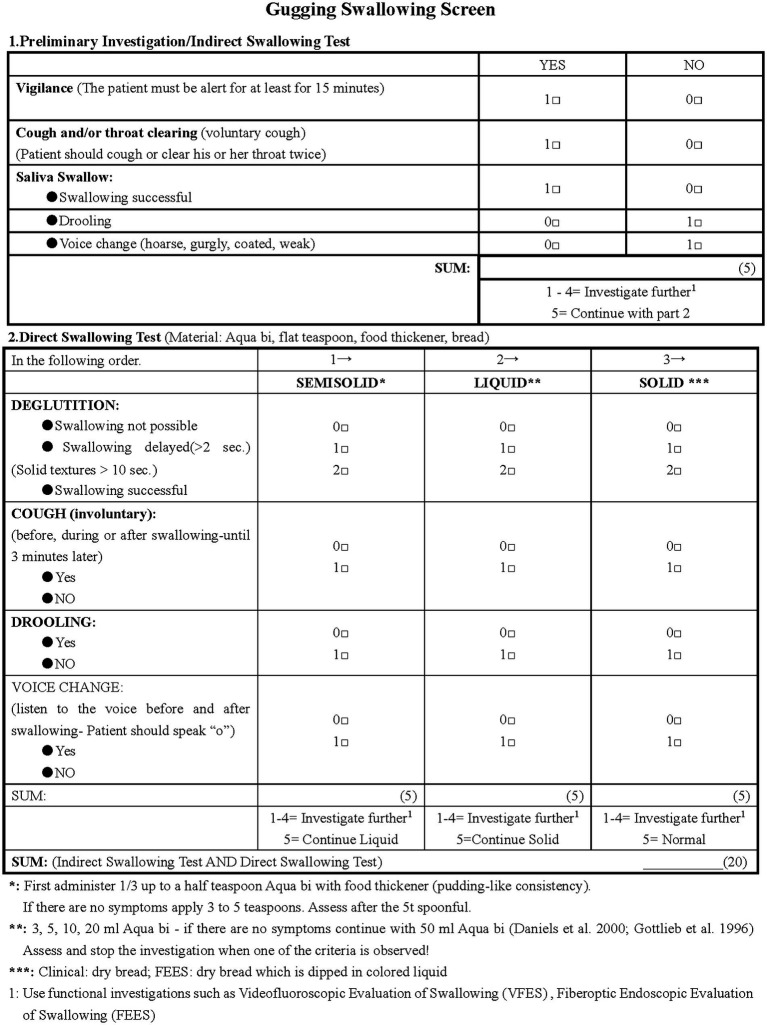
GUSS scores. GUSS, Gugging Swallowing Screen.

Dysphagia assessment was conducted using the Gugging Swallowing Screen (GUSS) score, which evaluates both indirect and direct swallowing tests (Figure 4) (17). Patients with a GUSS score of 20 points were classified as having normal swallowing function, while those with a score <20 points as having dysphagia (18).

### 2.3 Data collection

The primary outcome measure in this study was occurrence of dysphagia. Clinical data were retrieved from inquiring the patient’s history, encompassing demographic information, such as sex, age, body mass index, smoking history, hypertension history, hyperglycaemia history, leukocyte count, neutrophil count, arterial and venous oxygen partial pressures, as well as spirometry parameters, including forced expiratory volume in 1 s (FEV1), FEV1/forced vital capacity (FVC) ratio, and FEV1 predicted value.

### 2.4 Statistical analysis

Statistical analysis was conducted using SPSS 27.0.1.0 (IBM Corp., Armonk, NY, USA) and R 4.3.3 software (R Software for Statistical Computing, Vienna, Austria). Participants were randomly divided into a training cohort (*n* = 210) and a validation cohort (*n* = 90) at a 7:3 ratio using a random number table. Variables with a normal distribution were expressed as means ± standard deviations ($\bar{x}$±s) and compared using the t-test. Non-normally distributed variables were expressed as median (Q1, Q3) and compared using the Mann–Whitney U-test. Categorical data are expressed as cases (%) and analyzed using the chi-square test. The intraclass correlation coefficient (ICC) was used to assess the consistency of these ultrasound measurement index between the same physician and other similarly qualified physicians.

In the training cohort, a univariate logistic regression analysis was performed to identify variables significantly associated with dysphagia occurrence. Statistically significant variables identified in this analysis were incorporated into multifactorial logistic regression analysis to determine independent risk factors for dysphagia. Using the rms package in R software, we established a list of independent risk factors predicting dysphagia occurrence. Then, this list was utilized to construct a nomogram model for predicting dysphagia occurrence within the rms program package in R software. Predictive performance of the nomograms was evaluated using the consistency index (C-index) and calibrated with 1,000 bootstrap samples. For clinical applications, probability of dysphagia development was calculated for each patient based on the nomogram. Receiver operating characteristic (ROC) curve analysis determined the optimal threshold by maximising the Youden’s index (i.e., sensitivity + specificity - 1). The accuracy of the optimal threshold was assessed using sensitivity, specificity, predictive value, and likelihood ratio. Additionally, calibration, clinical decision, and clinical impact curves were generated to further evaluate the predictive efficacy, accuracy, and clinical utility of the model. The level of significance was set at *p* < 0.05.

## 3 Results

### 3.1 Comparison of basic characteristics data between the patient groups

Among the 300 patients with COPD included in our study, 155 (51.67%) developed dysphagia, while 145 (48.33%) did not. Notably, no significant differences were observed between the training and validation cohorts in any of the variables analyzed (*p* < 0.05) (Table 1).

**Table 1:** Basic characteristics of patients in the training and validation cohorts.

	Cohorts		
Characteristic	Training cohort (*n* = 210)	Validation cohort (*n* = 90)	*p*-value
Age (years)	67.79 ± 10.77	67.53 ± 10.92	0.853
Sex, *n* (%)			0.516
Male	128 (60.95%)	59 (65.56%)	
Female	82 (39.05%)	31 (34.44%)	
BMI (kg/m^2^)	22.61 ± 2.63	22.32 ± 2.56	0.380
Duration of COPD (years)	6.25 ± 2.16	6.56 ± 1.81	0.244
Smoking history, *n* (%)			
Yes	118 (55.91%)	54 (60.00%)	
No	92 (44.09%)	36 (40.00%)	
Hypertension history, *n* (%)			0.980
Yes	121 (57.62%)	52 (57.78%)	
No	89 (42.38%)	38 (42.22%)	
Diabetes history, *n* (%)			0.790
Yes	70 (33.33%)	32 (35.56%)	
No	140 (66.67%)	58 (64.44%)	
White blood cell count (×10^9^/L)	6.74 ± 2.17	6.59 ± 2.46	0.587
Neutrophil count (×10^9^/L)	54.92 ± 6.47	53.48 ± 6.85	0.083
Partial pressure of arterial oxygen (mmHg)	102.41 ± 6.76	102.44 ± 7.29	0.968
Partial pressure of venous oxygen (mmHg)	38.08 ± 2.68	38.76 ± 3.06	0.071
Carbon dioxide partial pressure (mmHg)	40.87 ± 5.78	40.62 ± 6.19	0.743
FEV1 (mL)	1732.63 ± 275.78	1728.80 ± 265.90	0.137
FEV1/FVC (%)	58.94 ± 4.73	58.99 ± 4.71	0.911
FEV1Pre (%)	65.00 ± 8.55	64.01 ± 9.08	0.933
COPD severity grading			0.896
Mild	5 (2.38%)	3 (3.33%)	
Moderate	198 (94.29%)	84 (93.34%)	
Severe	7 (3.33%)	3 (3.33%)	
Change in lingual muscle thickness (cm)	0.99 ± 0.34	0.98 ± 0.41	0.367
Hyoid displacement (cm)	1.57 ± 0.42	1.65 ± 0.40	0.853
DMG (cm)	1.68 ± 0.29	1.69 ± 0.32	0.160
TMG (s)	1.26 ± 0.40	1.28 ± 0.42	0.832
MHLA (cm)	2.92 ± 0.37	2.91 ± 0.35	0.63
NHLA (cm)	1.73 ± 0.30	1.72 ± 0.30	0.870
HLAS (cm)	1.22 ± 0.30	1.20 ± 0.33	0.564
ASR (%)	39.93 ± 7.47	39.55 ± 7.76	0.694
GUSS Score (points)	14.28 ± 6.56	15.26 ± 5.98	0.225

ASR, hyoid-larynx shortening rate; BMI, body mass index; DMG, distance of movement of the genioglossus; FEV1, forced expiratory volume in 1 s; FVC, forced vital capacity; GUSS, Gugging Swallowing Screen; HLAS, Hyoid-larynx shortening distance; MHLA, maximum hyoid-larynx approximation; NHLA, minimum hyoid-larynx approximation; TMG, time of movement of the genioglossus.

### 3.2 Development and validation of a nomogram for predictive modeling of dysphagia

The ICCs showed good consistency and reproducibility for change in lingual muscle thickness, hyoid displacement, DMG, TMG, MHLA, NHLA, HLAS and ASR (all ICC >0.7) measured by the same physician and different physicians with the same qualifications (Table 2).

**Table 2:** Consistency and reproducibility analysis within the same operator and among operators with the same qualifications.

Subjects	The same operator OR (95%CI)	Operators with the same qualifications OR (95%CI)
Change in lingual muscle thickness	0.878 (0.846–0.921)	0.742 (0.546–0.921)
Hyoid displacement	0.802 (0.726–0.871)	0.738 (0.546–0.891)
DMG	0.820 (0.716–0.931)	0.758 (0.620–0.842)
TMG	0.881 (0.766–0.943)	0.825 (0.764–0.943)
MHLA	0.834 (0.716–0.896)	0.772 (0.656–0.865)
NHLA	0.895 (0.736–0.928)	0.822 (0.646–0.892)
HLAS	0.903 (0.812–0.943)	0.760 (0.593–0.825)
ASR	0.814 (0.584–0.845)	0.752 (0.603–0.812)

ASR, hyoid-larynx shortening rate; CI, confidence interval; DMG, distance of movement of the genioglossus; HLAS, Hyoid-larynx shortening distance; MHLA, maximum hyoid-larynx approximation; NHLA, minimum hyoid-larynx approximation; OR, odds ratio; TMG, time of movement of the genioglossus.

Univariate analysis revealed that changes in lingual muscle thickness, hyoid displacement, distance of movement of the genioglossus (DMG), time of movement of the genioglossus (TMG), NHLA, HLAS, and ASR were identified as risk factors for dysphagia development (Table 3). Multifactorial analysis further demonstrated that DMG, TMG, HLAS, and ASR were independently associated with dysphagia (Table 4).

**Table 3:** Univariate regression analysis based on training cohort.

Subject	*β*-value	OR (95%CI)	*p*-value
Age, years	0.002	1.001 (0.975–1.026)	0.976
Sex, male or female	0.168	1.184 (0.677–2.068)	0.554
BMI (kg/m^2^)	0.008	1.008 (0.909–1.118)	0.883
Duration of COPD (years)	0.115	1.122 (0.986–1.277)	0.066
Smoking history, yes or no	0.133	1.142 (0.660–1.978)	0.635
Hypertension history, yes or no	0.238	1.269 (0.731–2.203)	0.397
Diabetes history, yes or no	0.265	1.303 (0.739–2.299)	0.361
White blood cell count (×10^9^/L)	0.077	1.080 (0.951–1.226)	0.235
Neutrophil count (×10^9^/L)	−0.002	0.998 (0.956–1.041)	0.913
Partial pressure of arterial oxygen (mmHg)	0.012	1.012 (0.972–1.054)	0.568
Partial pressure of venous oxygen (mmHg)	0.053	1.054 (0.951–1.168)	0.313
Carbon dioxide partial pressure (mmHg)	0.012	1.012 (0.965–1.061)	0.617
FEV1 (mL)	0.001	1.001 (1.000–1.002)	0.350
FEV1/FVC (%)	−0.004	0.996 (0.940–1.055)	0.885
FEV1Pre (%)	0.003	1.003 (0.972–1.036)	0.835
COPD severity grading			
Mild			0.983
Moderate	0.118	0.889 (0.086–9.162)	0.921
Severe	0.162	0.851 (0.139–5.203)	0.861
Change in lingual muscle thickness (cm)	−2.633	0.072 (0.027–0.191)	**<0.001**
Hyoid displacement (cm)	−1.624	0.197 (0.093–0.416)	**<0.001**
DMG (cm)	−8.708	0.001 (0.001–0.002)	**<0.001**
TMG (s)	3.067	21.664 (7.983–58.793)	**<0.001**
MHLA (cm)	0.306	1.357 (0.641–2.876)	0.425
NHLA (cm)	5.287	197.790 (42.554–919.312)	**<0.001**
HLAS (cm)	−5.018	0.007 (0.002–0.028)	**<0.001**
ASR (%)	−0.491	0.612 (0.534–0.701)	**<0.001**

ASR, hyoid-larynx shortening rate; BMI, body mass index; CI, confidence interval; DMG, distance of movement of the genioglossus; FEV1, forced expiratory volume in 1 s; FVC, forced vital capacity; HLAS, Hyoid-larynx shortening distance; MHLA, maximum hyoid-larynx approximation; NHLA, minimum hyoid-larynx approximation; OR, odds ratio; TMG, time of movement of the genioglossus. Bold value indicates statistically significant differences.

**Table 4:** Multi-factor logistic regression analysis based on training cohort.

Variable	*β*-value	OR (95%CI)	*p*-value
Change in lingual muscle thickness, cm	−1.737	0.176 (0.018–1.720)	0.135
Hyoid displacement, cm	−0.395	0.673 (0.139–3.264)	0.623
DMG, cm	−8.220	0.001 (0.000–0.023)	**<0.01**
TMG, s	2.475	11.895 (1.437–98.275)	**0.022**
NHLA, cm	3.773	43.495 (0.842–246.356)	0.061
HLAS, cm	−4.148	0.016 (0.001–0.275)	**0.004**
ASR, %	−0.508	0.601 (0.470–0.770)	**<0.01**

ASR, hyoid-larynx shortening rate; BMI, body mass index; CI, confidence interval; DMG, distance of movement of the genioglossus; FEV1, forced expiratory volume in 1 s; FVC, forced vital capacity; GUSS, Gugging Swallowing Screen; HLAS, Hyoid-larynx shortening distance; MHLA, maximum hyoid-larynx approximation; NHLA, minimum hyoid-larynx approximation; OR, odds ratio; TMG, time of movement of the genioglossus. Bold value indicates statistically significant differences.

**Figure 5:**
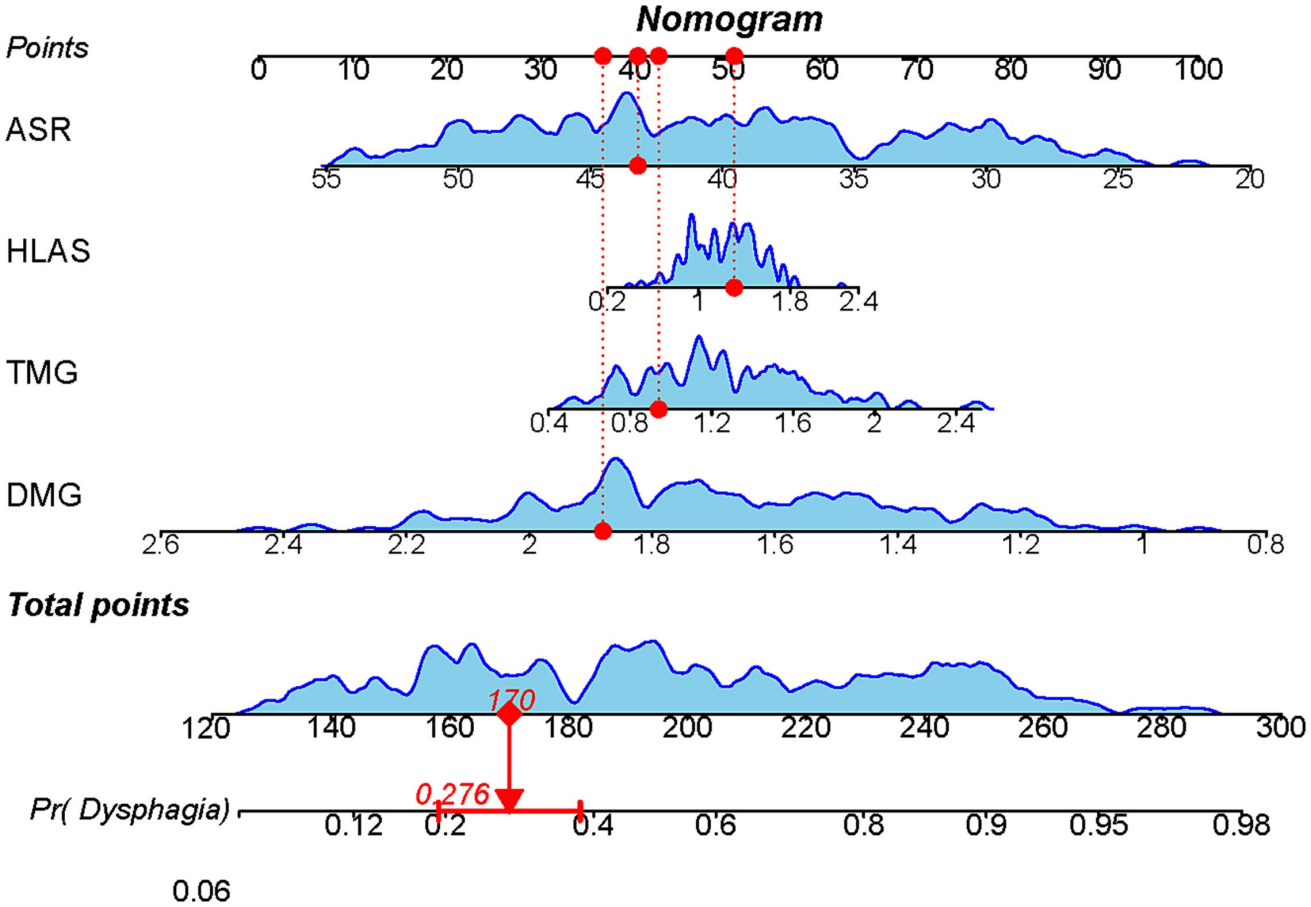
Nomogram model constructed based on ultrasound features to predict the occurrence of dysphagia. Each factor’s value on the horizontal axis is individually positioned to obtain the factor’s score. The score of each factor is then summed to obtain the total score, corresponding to the point on the axis representing the risk of dysphagia, i.e., the patient’s probability of experiencing dysphagia.

**Figure 6:**
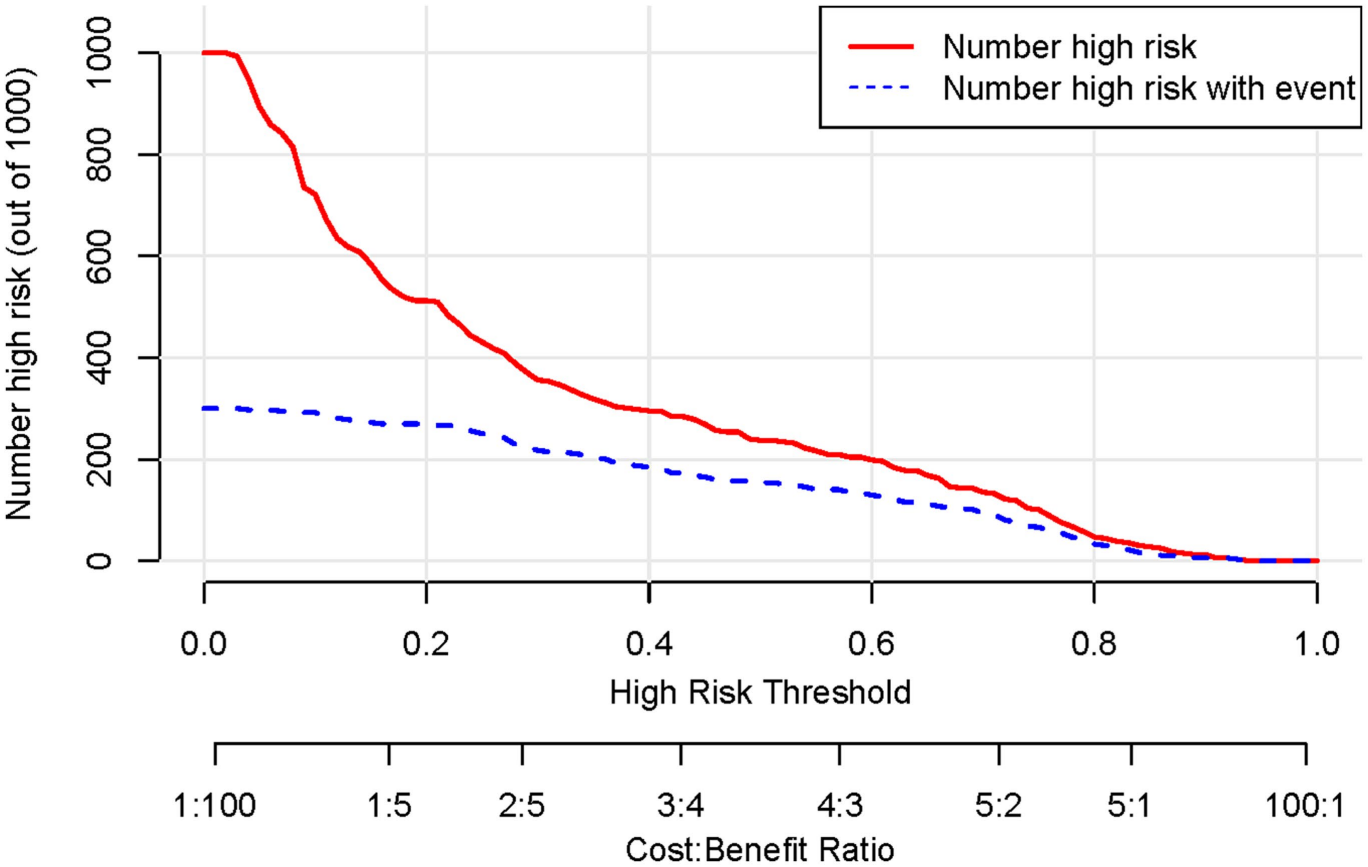
Receiver operating characteristics curves depicting the performance of the column-line graphical model in predicting onset of dysphagia in the training (*n* = 210) and validation cohorts (*n* = 90), respectively.

**Figure 7:**
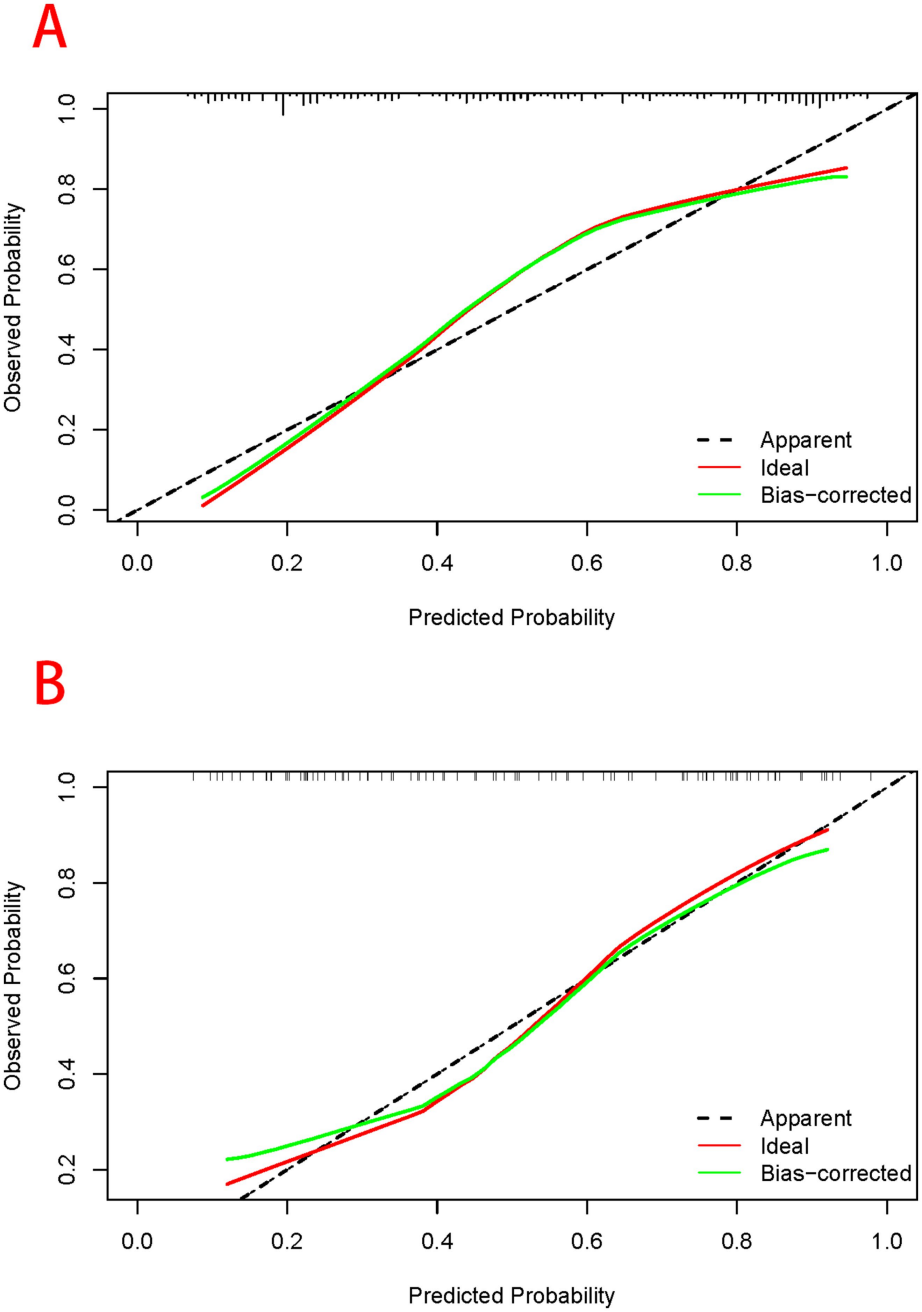
Assessment of predictive performance of the model when participants in the training **(A)** and validation cohorts **(B)** are at risk of developing dysphagia. The horizontal axis of the calibration curve represents the predicted probability of dysphagia calculated using the column-line diagram model, while the vertical axis represents the actual probability of dysphagia. The light blue line passing through the origin represents the ideal diagnostic result, while black solid line represents the prediction result of the model. The closer the predicted solid line of the model aligns to the ideal diagnostic result, the better the predictive performance of the model.

**Figure 8:**
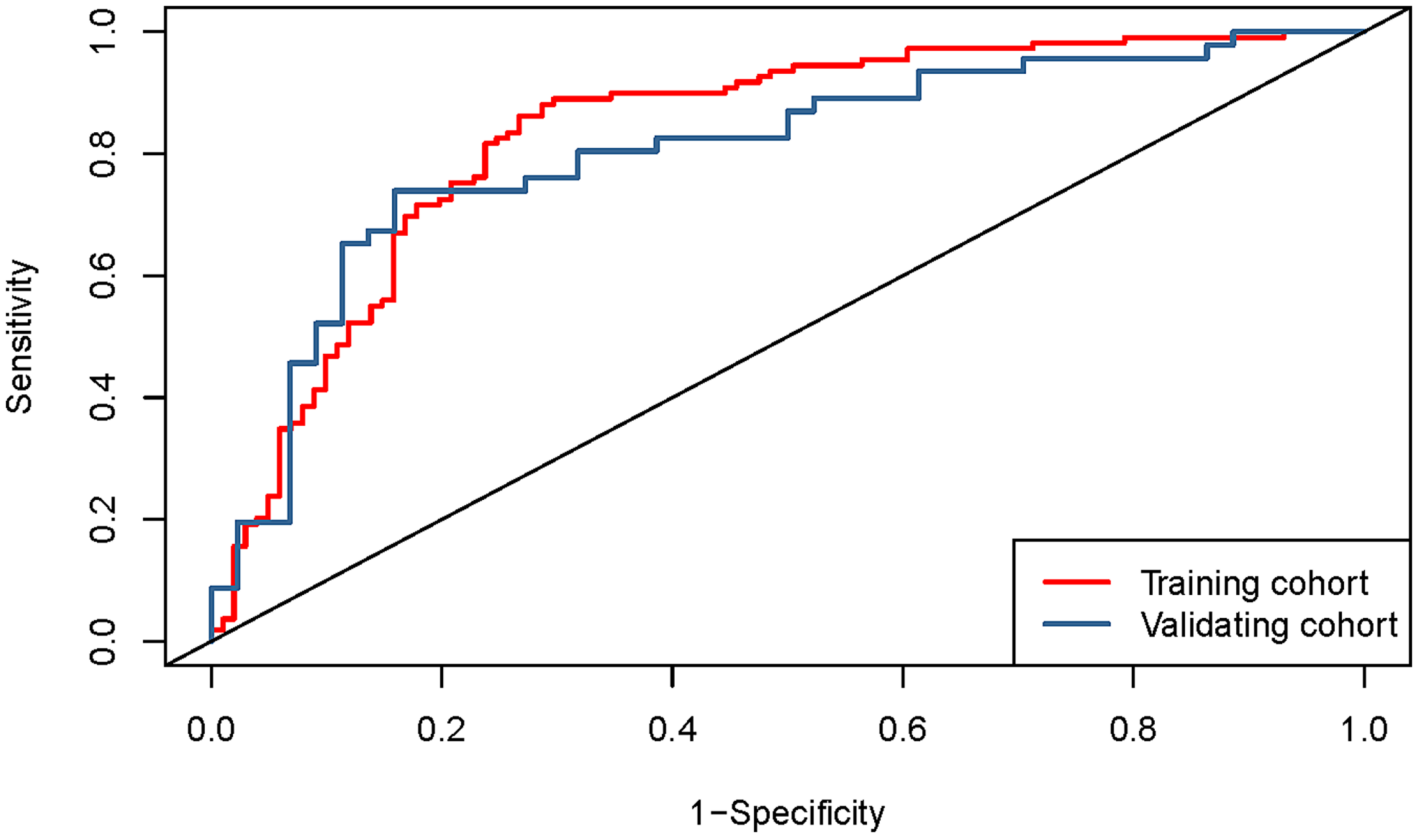
Decision curve of the nomogram model for predicting occurrence of dysphagia in patients with COPD. The horizontal axis represents the domain probability value, vertical axis represents the net benefit rate. The light blue curve represents the assumption that all patients with COPD develop dysphagia, while black straight line represents the assumption that all patients with COPD do not develop dysphagia. The red curve represents the columnar graphical model constructed in the present study, which predicts a high level of benefit for the development of dysphagia when the range of the domain probability is >8%. COPD, chronic obstructive pulmonary disease.

**Figure 9:**
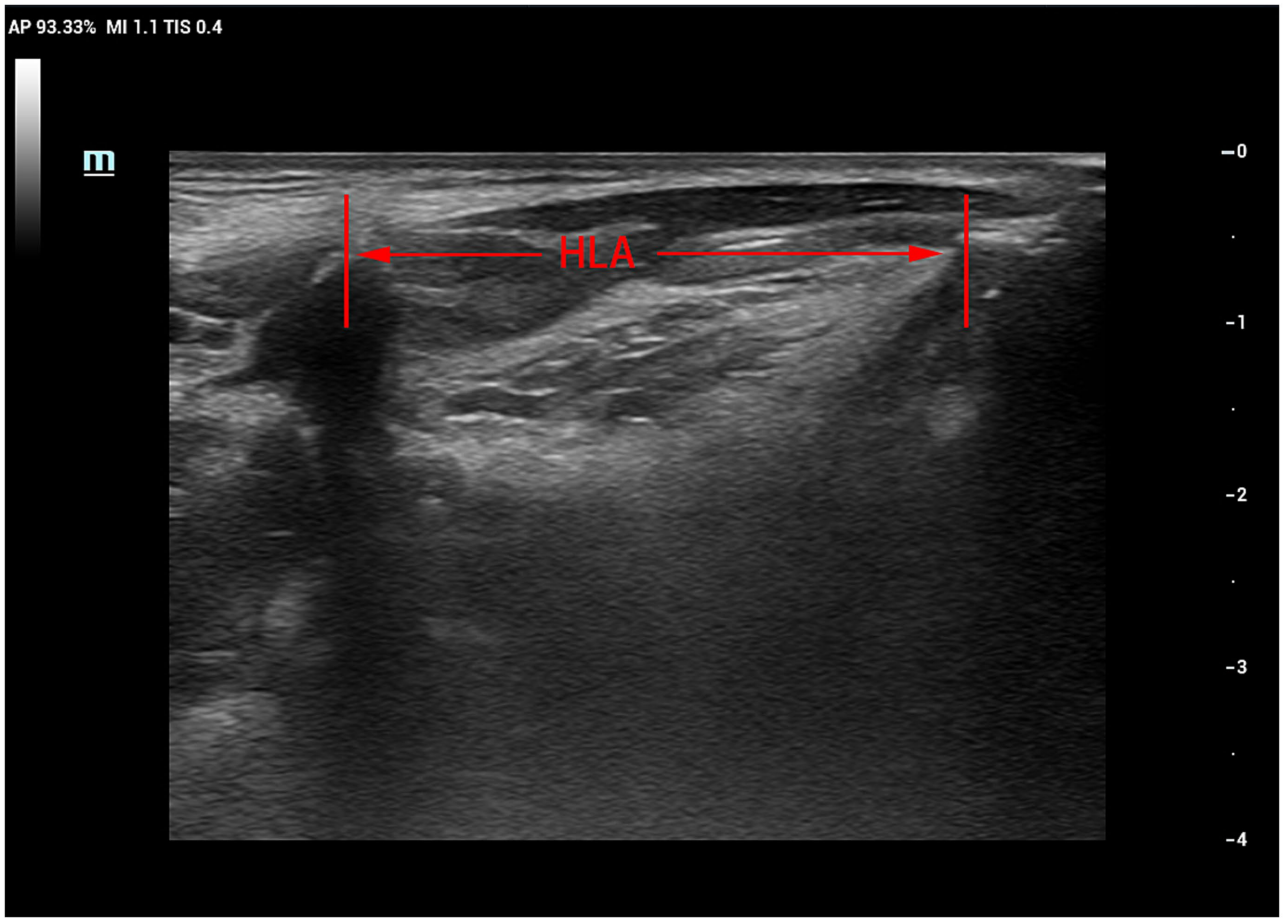
Clinical impact curves of a column-line graphical model predicting development of dysphagia in patients with COPD. COPD, chronic obstructive pulmonary disease.

Based on the results of the multifactorial logistic regression analysis, four risk factors—ASR, HLAS, TMG, and DMG—were integrated into R software to construct a nomogram model for dysphagia prediction. The final nomogram is shown in Figure 5. The area under the ROC curve (AUC) values of the nomogram model in the training and validation cohorts were 0.834 (95% confidence interval [CI], 0.777–0.890) and 0.804 (95%CI, 0.711–0.898), respectively, indicating good predictive efficacy (Figure 6). Calibration curves showed that the predicted probability of dysphagia in the training and validation cohorts aligned well with the actual probability, indicating high calibration (Figures 7A,B). Decision curve analysis (DCA) was used to evaluate the clinical utility of the model, and the results showed that patients in the training cohort benefited most when the predicted probability ranged from 12.7–82.7%, and those in the validation cohort benefited most when the probability ranged from 26.2–81.1%. This wide range of domain probabilities indicates the model’s clinical utility (Figure 8). Clinical impact curves showed that when the threshold probability exceeded 70% of the predicted score probability value, the ‘number of patients labeled as high-risk by the model’ line was very close to the ‘number of patients at risk’ line, indicating that the model accurately identified high-risk patients with dysphagia among those with COPD (19). These findings confirm the model’s high net clinical benefit (Figure 9).

### 3.3 Risk of dysphagia based on the nomogram score

The Hosmer–Lemeshow goodness-of-fit test coefficient for the column-line graphical model was 0.235. In the training and validation cohorts, the C-statistic values were 0.834 and 0.804, respectively. The best truncation probabilities were 0.439 and 0.544, sensitivities 0.862 and 0.739, specificities 0.733 and 0.841, positive predictive values 0.777 and 0.829, negative predictive values 0.831 and 0.755, positive likelihood ratios 3.226 and 4.646, and negative likelihood ratios 0.188 and 0.310, respectively (Table 5).

**Table 5:** Accuracy of assessing dysphagia using nomogram.

Parameter	Training cohort	Validating cohort
Area under the ROC	0.834 (0.777–0.890)	0.804 (0.711–0.898)
Hosmer-Lemeshow test	0.235	
Cut-off probability	0.439	0.544
Youden’s index	0.595	0.580
Sensitivity (%)	0.862	0.739
Specificity (%)	0.733	0.841
Positive predictive value (%)	0.777	0.829
Negative predictive value (%)	0.831	0.755
Positive likelihood ratio	3.226	4.646
Negative likelihood ratio	0.188	0.310

CI, confidence interval; OR, odds ratio; ROC, receiver-operating characteristic.

## 4 Discussion

Swallowing is a complex biomechanical process that is synchronized with respiration to safeguard the airway. In the population with COPD, abnormal breathing patterns characterized by a coordination imbalance between breathing and swallowing may be observed. In healthy individuals, swallowing interrupts the respiratory cycle during exhalation, with a respiratory pause lasting approximately 0.5 to 1.5 s, followed by breathing resumption typically in the exhalation phase after swallowing. However, in patients with COPD, the act of swallowing or resumption of breathing after swallowing often occurs during inhalation. This aberrant respiratory-swallowing pattern is closely linked to upper airway dysfunction, not only increasing the likelihood of swallowing impairments in patients with COPD but also potentially compromising airway safety, leading to aspiration (20–22). Furthermore, inflammation reactions may manifest in the airways and throat of patients with COPD, leading to swelling and discomfort in the throat mucosa, consequently impeding the seamless progression of swallowing. Hence, this intricately regulated process may not be compatible with individuals afflicted by COPD. Dysphagia in patients with COPD poses significant risks, including exacerbation of dyspnoea, malnutrition, reduced quality of life, and the potential for life-threatening aspiration events. VFSS is a specialized imaging procedure performed under X-ray fluoroscopy to evaluate the swallowing function of the oropharynx, larynx, and oesophagus. VFSS allows for a detailed assessment and analysis of the entire swallowing process. By observing lateral and anterior–posterior images, different stages of swallowing, including the oral preparatory, oral, pharyngeal, and oesophageal phases, can be evaluated. It also enables the observation of anatomical structures such as the tongue, soft palate, pharynx, and the transportation process of bolus. However, a systematic review and meta-analysis demonstrated that the sensitivity of VFSS was only 0.77, significantly lower than that of flexible endoscopic evaluation of swallowing (FEES) at 0.88 (23). FEES also has limitations, such as the inability to visualize the complete oral phase of swallowing and the risk of missing aspiration events during ‘whitening’ of the endoscope during pharyngeal contraction. Therefore, the use of FEES remains somewhat limited despite its advantages. In recent years, ultrasound has aroused widespread interest in the evaluation of swallowing function. Huang et al. (18) found that Hyoid muscle thickness, hyoid bone displacement, geniohyoid muscle movement distance, HLAS, and ASR were significantly correlated with swallowing dysfunction in COPD patients, and had good diagnostic effectiveness.

In our study, dysphagia was prevalent, affecting 155 (51.67%) patients. We identified ASR, HLAS, TMG, and DMG as independent risk factors for dysphagia in patients with COPD. These factors were integrated into an R software-based prediction model. We visualized it using a line graph to enhance its practicality. Subsequently, we rigorously evaluated and validated the model using various metrics, including the area under the ROC curve, calibration curve, DCA, and clinical impact curve, ensuring its reliability and applicability in clinical practice.

The ASR, a widely recognized predictor of dysphagia, its relationship to swallowing dysfunction lies in its ability to indicate the effectiveness of laryngeal closure, which is crucial for preventing aspiration during the swallowing process. (18, 24), likely holds its predictive power owing to its impact on tongue movement dynamics. Shortening the distance between the hyoid bone and thyroid cartilage, as indicated by a higher ASR, can disrupt smooth flow of food or liquid through the oesophagus and increase the risk of accidental aspiration. Similarly, elevated HLAS may restrict hyoid bone movement, impairing the coordination and efficiency of the oropharyngeal phase. The hyoid bone is a key structure in the process of swallowing and is involved in the pushing and coordination of food. Restricted hyoid movement may result in uncoordinated or incomplete swallowing movements. Besides, an elevated HLAS may potentially lead to discomfort during swallowing and reducing swallowing efficiency. This could result in patient aversion to swallowing, affecting their dietary intake and overall quality of life. In addition, our study identified excessive genioglossus muscle movement time and insufficient genioglossus muscle movement distance as independent risk factors for dysphagia in patients with COPD. Prolonged muscle movement time may signify slow execution of swallowing manoeuvres, leading to delayed or difficult swallowing. Conversely, inadequate movement distance of chin and tongue muscles may impede effective food or liquid propulsion, increasing the risk of dysphagia or aspiration. It is worth noting that single-factor grading in this study did not show statistical significance. This may be because this study included patients with no previous history of acute exacerbations, and the COPD severity grading in these patients was relatively mild. None of the included patients had extremely severe COPD.

In this study, we developed a prediction model incorporating patient baseline characteristics, lung function parameters, and ultrasound indices. Through comprehensive evaluation using diverse methods, the model demonstrated robust reliability. Presenting the model as a line graph enhances its intuitiveness, flexibility, and applicability in clinical settings. This facilitates prompt identification of patients with COPD at risk of dysphagia, enabling healthcare providers to promptly initiate timely therapeutic interventions, such as dietary modifications and swallowing training, to enhance swallowing function.

This study had some limitations. First, it was conducted at a single centre with a relatively small sample size, warranting external validation in larger, multicentre cohorts for broader applicability. Second, the scope of included imaging and clinical laboratory tests was limited, potentially overlooking certain risk factors related to dysphagia. Subsequent studies should expand on these findings by incorporating additional relevant variables to refine the model. By further expanding patient data and screening for indicators with a higher correlation with dysphagia, future studies can enhance the predictive accuracy and clinical utility of the model. Third, it mainly employed the GUSS score as the diagnostic criterion. In future research endeavors, the incorporation of more comprehensive and precise diagnostic methods such as VFSS or FESS could be contemplated to further validate and enrich the research outcomes. Furthermore, the lack of a healthy control group for comparison presents a significant limitation in our study. Inclusion of a healthy control group in future research could provide a more comprehensive understanding of the differences between individuals with COPD and those without the condition.

In conclusion, we utilized four key risk factors (ASR, HLAS, TMG and DMG) identified using multifactorial regression analysis to construct a robust predictive model for dysphagia. This nomogram model offers an improved framework for assessing dysphagia risk in patients with COPD.

## Data Availability

The raw data supporting the conclusions of this article will be made available by the authors, without undue reservation.

## References

[1] Christenson SA Smith BM Bafadhel M Putcha N. Chronic obstructive pulmonary disease. Lancet. (2022) 399:2227–42. doi: 10.1016/S0140-6736(22)00470-6, PMID: 35533707

[2] Cvejic L Bardin PG. Swallow and aspiration in chronic obstructive pulmonary disease. Am J Respir Crit Care Med. (2018) 198:1122–9. doi: 10.1164/rccm.201804-0704PP, PMID: 29939762

[3] Broers C Tack J Pauwels A. Review article: gastro-oesophageal reflux disease in asthma and chronic obstructive pulmonary disease. Aliment Pharmacol Ther. (2018) 47:176–91. doi: 10.1111/apt.14416, PMID: 29193245

[4] Thiyagalingam S Kulinski AE Thorsteinsdottir B Shindelar KL Takahashi PY. Dysphagia in older adults. Mayo Clin Proc. (2021) 96:488–97. doi: 10.1016/j.mayocp.2020.08.001, PMID: 33549267

[5] Wang X Lu J Song Z Zhou Y Liu T Zhang D. From past to future: bibliometric analysis of global research productivity on nomogram (2000-2021). Front Public Health. (2022) 10:997713. doi: 10.3389/fpubh.2022.997713, PMID: 36203677 PMC9530946

[6] Wong SM Domangue RJ Fels S Ludlow CL. Evidence that an internal schema adapts swallowing to upper airway requirements. J Physiol. (2017) 595:1793–814. doi: 10.1113/JP272368, PMID: 27883179 PMC5330896

[7] Bolser DC Pitts TE Davenport PW Morris KF. Role of the dorsal medulla in the neurogenesis of airway protection. Pulm Pharmacol Ther. (2015) 35:105–10. doi: 10.1016/j.pupt.2015.10.012, PMID: 26549786 PMC4690802

[8] Spilling CA Jones PW Dodd JW Barrick TR. White matter lesions characterise brain involvement in moderate to severe chronic obstructive pulmonary disease, but cerebral atrophy does not. BMC Pulm Med. (2017) 17:92. doi: 10.1186/s12890-017-0435-1 28629404 PMC5474872

[9] Regan J Lawson S De Aguiar V. The eating assessment Tool-10 predicts aspiration in adults with stable chronic obstructive pulmonary disease. Dysphagia. (2017) 32:714–20. doi: 10.1007/s00455-017-9822-2, PMID: 28707015

[10] Yoshimatsu Y Tobino K Sueyasu T Nishizawa S Ko Y Yasuda M. Repetitive saliva swallowing test predicts COPD exacerbation. Int J Chron Obstruct Pulmon Dis. (2019) 14:2777–85. doi: 10.2147/COPD.S226268, PMID: 31824143 PMC6900275

[11] Salam S Allen J Dimachkie MM Hanna MG Machado PM. Imaging swallowing function and the mechanisms driving dysphagia in inclusion body myositis. Clin Exp Rheumatol. (2024) 42:425–35. doi: 10.55563/clinexprheumatol/t1x3qa, PMID: 38372730

[12] Arai N Hanayama K Yamazaki T Tomita T Tsubahara A Sugamoto K. A novel fluoroscopic method for multidimensional evaluation of swallowing function. Auris Nasus Larynx. (2019) 46:83–8. doi: 10.1016/j.anl.2018.04.005, PMID: 29753584

[13] Allen JE Clunie GM Winiker K. Ultrasound: an emerging modality for the dysphagia assessment toolkit? Curr Opin Otolaryngol Head Neck Surg. (2021) 29:213–8. doi: 10.1097/MOO.0000000000000708, PMID: 33741822 PMC7611059

[14] Zhang Z Perera S Donohue C Kurosu A Mahoney AS Coyle JL. The prediction of risk of penetration-aspiration via hyoid bone displacement features. Dysphagia. (2020) 35:66–72. doi: 10.1007/s00455-019-10000-5, PMID: 30919104 PMC6764901

[15] Hsiao MY Chang YC Chen WS Chang HY Wang TG. Application of ultrasonography in assessing oropharyngeal dysphagia in stroke patients. Ultrasound Med Biol. (2012) 38:1522–8. doi: 10.1016/j.ultrasmedbio.2012.04.017, PMID: 22698507

[16] Agustí A Celli BR Criner GJ Halpin D Anzueto A Barnes P. Global initiative for chronic obstructive lung disease 2023 report: gold executive summary. Eur Respir J. (2023) 61:232–48. doi: 10.1183/13993003.00239-2023, PMID: 36858443 PMC10066569

[17] Trapl M Enderle P Nowotny M Teuschl Y Matz K Dachenhausen A. Dysphagia bedside screening for acute-stroke patients: the Gugging swallowing screen. Stroke. (2007) 38:2948–52. doi: 10.1161/STROKEAHA.107.483933, PMID: 17885261

[18] Huang Y Zhong H Xu Z Su Q Su S. Assessing swallowing dysfunction aggravation in chronic obstructive pulmonary disease patients using ultrasonic measurements with swallowing movement parameters. J Ultrasound Med. (2024) 43:501–11. doi: 10.1002/jum.16381, PMID: 38009681

[19] Zhong H Liu Y Liu P Wang Z Lian X Xu Z. Risk estimation for postoperative nausea and vomiting: development and validation of a nomogram based on point-of-care gastric ultrasound. BMC Anesthesiol. (2023) 23:393. doi: 10.1186/s12871-023-02345-0, PMID: 38036983 PMC10688051

[20] Drulia T Hodge A. Clinical practice patterns of speech-language pathologists delivering dysphagia services to persons with COPD: analysis of survey outcomes. Semin Speech Lang. (2021) 42:363–83. doi: 10.1055/s-0041-1735846, PMID: 34729725

[21] Gross RD Atwood CW Ross SB Olszewski JW Eichhorn KA. The coordination of breathing and swallowing in chronic obstructive pulmonary disease. Am J Respir Crit Care Med. (2009) 179:559–65. doi: 10.1164/rccm.200807-1139OC, PMID: 19151193

[22] Pinto CF Balasubramanium RK Acharya V. Nasal airflow monitoring during swallowing: evidences for respiratory-swallowing incoordination in individuals with chronic obstructive pulmonary disease. Lung India. (2017) 34:247–50. doi: 10.4103/lungindia.lungindia_117_16, PMID: 28474650 PMC5427752

[23] Giraldo-Cadavid LF Leal-Leaño LR Leon-Basantes GA Bastidas AR Garcia R Ovalle S. Accuracy of endoscopic and videofluoroscopic evaluations of swallowing for oropharyngeal dysphagia. Laryngoscope. (2017) 127:2002–10. doi: 10.1002/lary.26419, PMID: 27859291

[24] Mepani R Antonik S Massey B Kern M Logemann J Pauloski B. Augmentation of deglutitive thyrohyoid muscle shortening by the Shaker exercise. Dysphagia. (2009) 24:26–31. doi: 10.1007/s00455-008-9167-y, PMID: 18685891 PMC2892888

